# The Era of Smartphones: Back to Our Biological Makeup?

**DOI:** 10.2196/mhealth.5193

**Published:** 2016-07-27

**Authors:** Alejandro Santos-Lozano, Nuria Garatachea, Helios Pareja-Galeano, Carmen Fiuza-Luces, Fabian Sanchis-Gomar, Alejandro Lucia

**Affiliations:** ^1^European University Miguel de CervantesGIDFYS, Department of Health Sciences.ValladolidSpain; ^2^Research Institute of Hospital 12 de Octubre (“i+12”)MadridSpain; ^3^Faculty of Health and Sport Sciences, University of ZaragozaHuesca, SpainSpain; ^4^European UniversityMadridSpain

Physical inactivity is a major modifiable cardiovascular risk factor that has become a growing health problem in the 21st century: 83% of adolescents aged 13-15 years and approximately 1/3 of adults worldwide are inactive, that is, not meeting the minimum international physical activity (PA) recommendations (≥150 minutes/week of moderate to vigorous PA) [1,2]. Thus, the PA levels of the general population, especially of individuals at cardiovascular risk, should be routinely assessed by health care professionals, as it has been recently recommended by the American Heart Association [3]. To this end, accelerometers (usually attached to an elastic belt around the waist) allow objective quantification of PA by providing continuous recordings. At least 3 to 5 days of accelerometer monitoring (including weekend days) are required to determine habitual PA, and it is generally accepted that the device should be worn for ≥10 hours/day [4]. For this reason, the simple and inexpensive method of PA questionnaires is more widely used and generally better accepted. Unfortunately, the validity of self-reported PA is questionable.

As recently discussed by Direito and collaborators [1], a reliable and simple strategy for assessing individual PA levels, without interfering with people’s daily life, is the use of smartphone apps. Smartphones are used by millions of people and many versions include a triaxial accelerometer and a positioning system among other types of sensors, thereby allowing the development of new apps with biomedical applicability. Other devices like the “Nike+ Move” app, which converts the smartphone into an “intelligent band,” or the Apple Watch and the HealthKit, might represent the beginning of the wearable’s revolution in health sciences, providing a great chance to monitor PA in an effective and inexpensive manner.

We have evolved to perform high levels of PA (>2-3 hours) on a daily basis as persistent hunter-gatherers. However, technological improvements over few generations (industrial and, most recently, digital revolution) have led to dramatic reductions in our PA levels leading to chronic maladaptation and disease, with prolonged television viewing exemplifying a behavior that is at odds with our biological makeup and is associated with an increased risk of mortality [5]. Let us hope that the technological improvements that have made us become so inactive might pay off one day by helping increase our PA levels, that is, through the use of smartphones and wearable devices.

## Abbreviations

PA: physical activity
